# Early specializations for mimicry and defense in a Jurassic stick insect

**DOI:** 10.1093/nsr/nwaa056

**Published:** 2020-04-02

**Authors:** Hongru Yang, Chaofan Shi, Michael S Engel, Zhipeng Zhao, Dong Ren, Taiping Gao

**Affiliations:** College of Life Sciences and Academy for Multidisciplinary Studies, Capital Normal University, Beijing 100048, China; School of Earth Sciences and Engineering, Guangdong Provincial Key Lab of Geodynamics and Geohazards, Guangdong Provincial Key Laboratory of Mineral Resources & Geological Processes, Sun Yat-sen University, Guangzhou 510275, China; Division of Entomology, Natural History Museum, and Department of Ecology & Evolutionary Biology, University of Kansas, Lawrence, KS 66045, USA; Division of Invertebrate Zoology, American Museum of Natural History, New York, NY 10024, USA; College of Life Sciences and Academy for Multidisciplinary Studies, Capital Normal University, Beijing 100048, China; College of Life Sciences and Academy for Multidisciplinary Studies, Capital Normal University, Beijing 100048, China; College of Life Sciences and Academy for Multidisciplinary Studies, Capital Normal University, Beijing 100048, China

**Keywords:** Susumanioidea, phylogeny, evolution, abdominal extension, antipredator strategy

## Abstract

Mimicry and secondary defense are staples among predator–prey interactions. Among insects, the stick and leaf insects are masters of camouflage. Nonetheless, a meager understanding of their origin and early mimetic evolution persists. Here, we report the earliest mimetic and defensive strategies of a stick insect from the Middle Jurassic of China, *Aclistophasma echinulatum* gen. et sp. nov., exquisitely preserving abdominal extensions and femoral spines. The distribution of these characteristics mapped onto the phylogeny of Phasmatodea reveals that abdominal extensions and femoral spines developed multiple times during the evolution of stick insects, and indicates that the origin of abdominal extensions predates other modifications, while tergal extensions predate other expansions of the body, such as those of the sterna and pleura, as well as defensive femoral spines. The new fossil provides clues into early antipredator defensive strategies, allows inferences as to the potential environment and predators, and reveals the mimetic and defensive mechanisms of stick insects from 165 million years ago.

## INTRODUCTION

Antipredator defenses among insects commonly involve the interplay of two functional categories [1,2]. The primary defense, also called passive defense, is the prey's avoidance of detection by the predator, usually by means of hiding or shifting periods of activity, crypsis, aposematism or pseudaposematism. The prey's secondary defense is evading capture after the initiation of a predator's attack. Secondary defenses involve active escape, antipredator displays, flash coloration, defensive chemical secretion and feigning death [1,2]. The active fighting of the prey against a predator when seized is sometimes referred to as a separate, third category [3,4]. But, together with the previous series of behaviors, they are referred to as active defenses [5]. Naturally, the ideal situation for any prey is to invest sufficiently in passive mechanisms of defense to avoid the chances of requiring active defense and the increased probability of death that comes when an attack has been initiated.

Phasmatodea, commonly referred to as walking sticks, stick and leaf insects, are icons of crypsis and primary defense specialization, exhibiting a wide range of remarkable morphological and behavioral modifications associated with camouflage [3,6,7]. The mimicry of extant stick and leaf insects may pervade all stages of life, from eggs resembling seeds for collection by ants, to nymphs mimetic with ants or scorpions and ultimately to the adults whose specialized morphology often blends them into the surrounding vegetation and even includes behaviors to mimic the swaying of twigs or leaves in the wind [3,8,9]. Phasmatodea deploy diverse defensive strategies involving both of the two aforementioned functional categories [3]. Aside from their (i) procryptic resemblance to sticks, leaves, moss or lichen, Phasmatodea have been observed in the field or lab to (ii) mimic ants or scorpions [8,9]; (iii) dropping [10], jumping [11] or taking flight to escape when disturbed [2]; (iv) producing a startling visual display by suddenly raising the tegmina and flashing bright colors or patterns on the hindwings, which also gives a supplementary effect through increasing the apparent size of the insect [12]; (v) producing disruptive sounds by rubbing the tegmina against the remigium [13] or rubbing a row of tubercles on one antenna against that of the other [14]; (vi) enacting thanatosis through catalepsy [15]; (vii) spraying defensive secretions [16–18] or releasing an odor [19]; and finally (viii) activating counterattacks by striking the metathoracic legs together and stabbing or impaling their aggressor with the hind femoral spines [1].

Fossil evidence of phasmatodean-associated mimicry and defensive behaviors is seldom documented [20,21]. Twig mimesis was reported in the Cretaceous stick-insect nymph *Elasmophasma stictum*, a species that exhibited traces of multiple extensions of the abdominal tergum, perhaps enhancing of the overall crypsis [20]. More dramatic, however, was the discovery of a leaf-mimicking species of Phylliinae from the middle Eocene, representing the earliest evidence of the leaf insects [21]. Nonetheless, the vast evolutionary diversity of the Phasmatodea, which at least extends into the Jurassic, is insufficiently known and the history of their mimetic and defensive behaviors remains unclear.

Herein, a new genus and species of Susumanioidea, *Aclistophasma echinulatum* gen. et sp. nov., is described from the Middle Jurassic of northeastern China. The new species exhibits a combination of characteristics associated with both passive and active defense mechanisms, such as abdominal extensions, femoral spines and large fore- and hindwings. The presence of these characteristics implies a remarkably early evolution of such specializations and their associated functions among some of the earliest Phasmatodea, suggesting that these defining and iconic aspects of stick-insect evolution appeared early and are among taxa that are otherwise considered as stem groups to the more typical members (Timematodea + Euphasmatodea) of the order today.

## RESULTS AND DISCUSSION

### Systematic paleontology

Order Phasmatodea Jacobson & Bianchi, 1902

Superfamily Susumanioidea Gorochov, 1988

Family Susumaniidae Gorochov, 1988

#### Aclistophasmatinae Yang, Engel & Gao subfam. nov.

##### Type genus.

*Aclistophasma* Yang, Engel & Gao gen. nov.

##### Diagnosis.

Forewing, RP with several branches; forking of M proximal to origin of RP; MP approaching CuA+CuPaα near middle. Hindwing, RP fused with MA near mid-length; CuP fused with 1A medially.

##### Remarks.

*Aclistophasma* differs from *Phasmomimoides* and *Adjacivena* by the presence of two branches to RP; from *Cretophasmomima* by the three branches of CuA+CuPaα; from *Eoprephasma, Renphasma, Orephasma, Aethephasma, Hagiphasma, Susumania, Kolymoptera, Prosusumania, Coniphasma* and *Eosusumania* by the forking of M proximal to origin of RP. *Palaeopteron, Promastacoides, Phasmomimella, Cretophasmomimoides, Phasmomimula* and *Paraphasmomimella* are known only from some preserved fragments of wings and cannot be meaningfully compared with this or many other taxa

#### *Aclistophasma* Yang, Engel & Gao gen. nov.

##### Type species.

*Aclistophasma echinulatum* Yang, Engel & Gao sp. nov.

##### Etymology.

The new generic name is a combination of the Greek aklystos (κλυστος, meaning ‘sheltered’, as in hidden and safe), ‘*aclist-*’ (meaning ‘crypsis’), *phasma* (meaning ‘spirit’) and the stem of the ordinal name Phasmatodea. The gender of the name is neuter.

##### Diagnosis.

Forewing: RP with two long branches; CuA fused with CuPaα apically; CuPaβ and CuPb fused proximally. Hindwing: RP with one or two branches; CuA fused with CuP apically. Edge of femora with spines. Abdominal terga extended, with minute spines or spicules on margins; abdominal segment X cleft medially; thorn pads present; male genitalia with vomer.

#### *Aclistophasma echinulatum* Yang, Engel & Gao sp. nov.

##### Etymology.

The epithet is from the Latin word ‘*echinulatus*’ (meaning ‘echinulate’).

##### Holotype.

Adult male, No. CNU-PHA-NN2019006; deposited in the fossil insect collection of Capital Normal University, Beijing.

##### Type locality and horizon.

The specimen was collected from the Middle Jurassic deposits of the Jiulongshan Formation near Daohugou Village, Ningcheng County, Inner Mongolia, China, which was dated around 165 Ma [7,22,23].

##### Diagnosis.

As for the genus (above).

##### Description.

**Figure 1.**
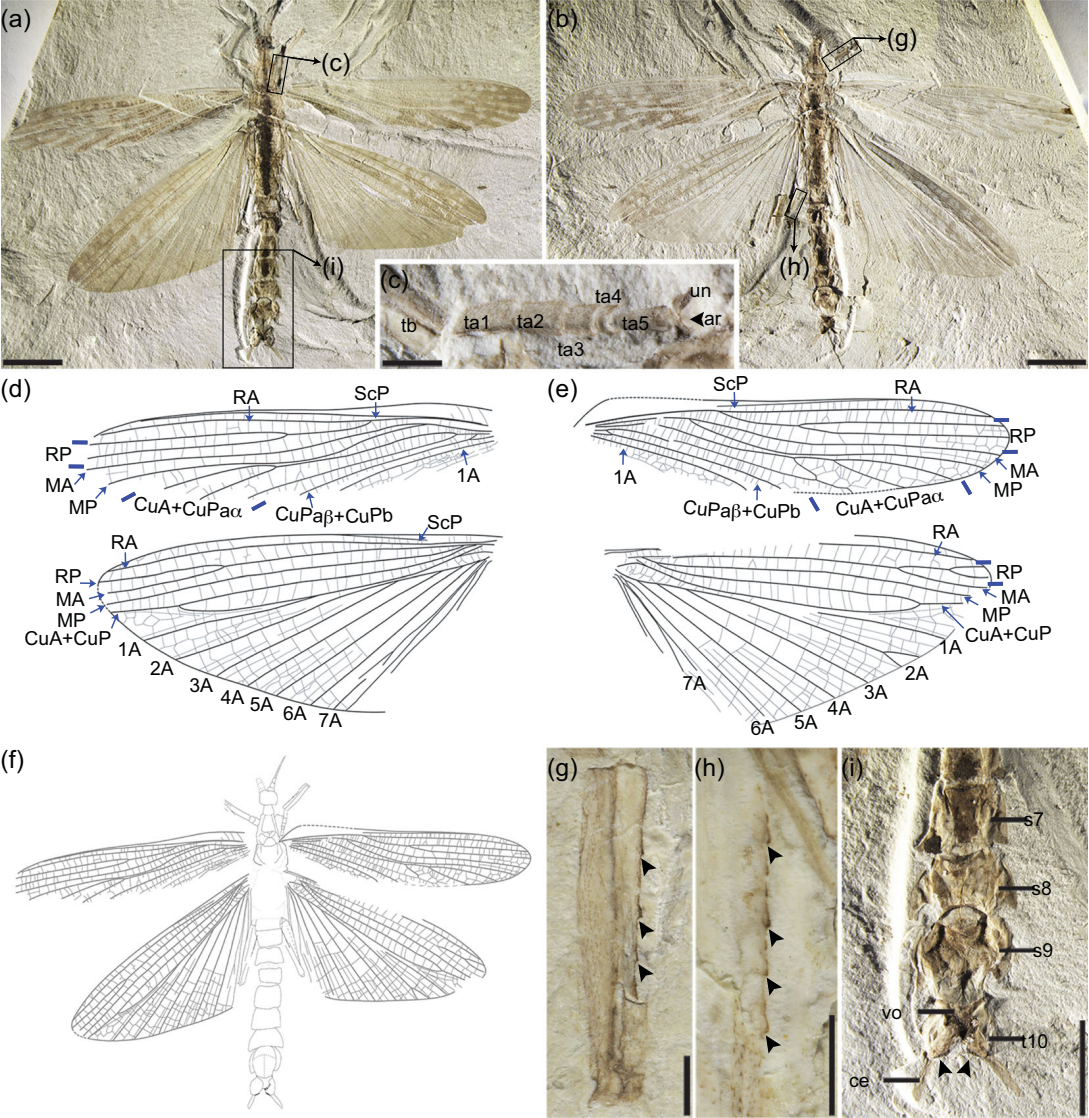
*Aclistophasma echinulatum* gen. et sp. nov., photographs of holotype CNU-PHA-NN2019006. (a) Part as preserved. (b) Counterpart as preserved. (c) Foretarsus. (d) Left fore- and hindwings. (e) Right fore- and hindwings. (f) Line drawing of part. (g) Forefemur of counterpart showing spines. (h) Hind femur of counterpart showing spines. (i) Male genitalia of part, with thorn pads indicated (black arrows). ar, arolium; ce, cercus; s7–9, sterna 7–9; t10, tergum 10; ta1–5, tarsomeres I–V; un, ungues; vo, vomer. Scale bars: (a) and (b), 10 mm; (c), (g) and (h), 1 mm; (i) 5, mm.

Fully winged male; whole body covered with numerous setae; head ovoid; antenna filiform, incompletely preserved; scape cylindrical, slightly shorter than wide; pedicel cylindrical, longer and thinner than scape; first flagellomere shorter than scape and pedicel combined; remaining flagellomeres incompletely preserved (Fig. 1a, b and f).

Pronotum trapezoidal, longer than wide; a distinctly transverse furrow on anterior part of pronotum; meso- and metathorax slightly wider and shorter than prothorax; metasternum distinctly separated from abdominal sternum I (Fig. 1a, b and f).

Forewings nearly complete; without ‘procostal’ area; area between costal margin and ScP wide in proximal part; ScP extending to two-thirds forewing length, parallel and close to RA; RA simple, straight; RP with two branches, forked a mid-length of forewing; MA and MP each simple; MP approaching CuA+CuPaα near middle; CuA with three branches fused with CuPaα apically; CuPaβ and CuPb fused proximally, simple and straight; an anal vein present, anal area wide and with many cross-veins (Fig. 1d and e).

Hindwing almost completely preserved; area between costal margin and ScP narrow; ScP terminating on costal margin at one-third of wing length; RA simple and straight, nearly reaching wing apex; RP fused with MA near mid-length; MP simple, extending to wing apex; CuA simple, fused with CuP apically; CuP simple and fused with 1A proximally; 2A–7A with a common origin near wing base; other anal veins indistinct (Fig. 1d and e).

Fore, middle and hind legs incompletely preserved; all femora with spines on both edges; dorsal carinae distinct on femora, with numerous tiny spines; profemur straight; right protibia folded against profemur; metafemur longer than profemur; tarsi pentamerous; basitarsus long but shorter than combined lengths of remaining tarsomeres; tarsomere IV shorter than others; unguis and arolia present, arolia large and of similar length to unguis (Fig. 1c, g and h).

**Figure 2.**
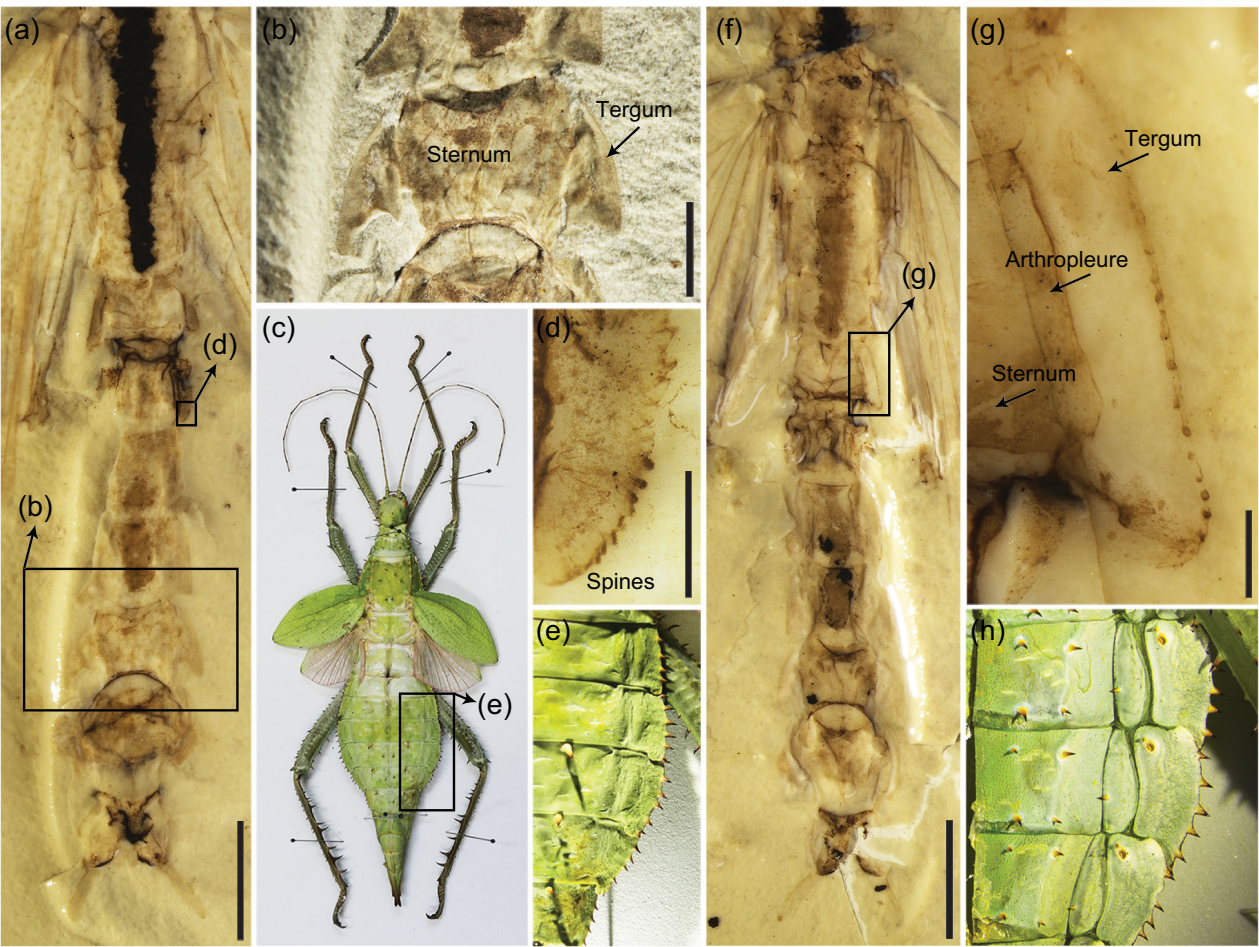
Abdominal extension of *Aclistophasma echinulatum* compared with extant stick insect. (a) Abdomen of part. (b) Abdominal segments VII–VIII of part. (c) Female of *Heteropteryx dilatata*. (d) Abdominal segment V, showing spines of abdominal tergal extension. (e) Detail of abdominal terga (dorsal view) of *H. dilatata*, showing extensions and spines. (f) Abdomen of counterpart. (g) Abdominal tergal extension of segment IV. (h) Detail of abdominal sterna (ventral view) of *H. dilatata*, showing abdominal extensions. Scale bars: (a) and (f), 10 mm; (b), 2 mm; (d) and (g), 0.5 mm.

Abdomen distinctly narrower than thorax, with 10 segments preserved; indistinct trace of alimentary canal straight; abdominal segments I–X with clearly extended terga, each tergum with tiny spines or spicules on margin and curved tip; abdominal segment I shortest of segments and same width as thorax; abdominal segments II–IV shorter than V–IX; abdominal segment V narrow proximally and separate from abdominal segment V; abdominal segment X narrow, apex cleft, forming two lobes medially, thorn pads present on inside of hind margin; subgenital plate splitting into two parts transversally, apical rounded and with longitudinal carina, extending to base of segment X; vomer present; cerci cylindrical, undivided and with numerous setae (Figs 1a, b and f, and 2a and f).

Measurements (in mm): body 56.18 (excluding antennae); head 3.09; antenna 5.64 (as preserved); scape 0.55; pedicel 0.64; flagellomeres I 0.61; prothorax 5.36; mesothorax 6.82; metathorax 6.73; forewing 43.55; hindwing 40.82; abdomen 32.91; profemur 6.18; protibia 5.36; protarsus 4.73; mesofemur 6.94; metafemur 10.64.

### Phylogenetic positions of *Aclistophasma echinulatum* and Susumanioidea

**Figure 3.**
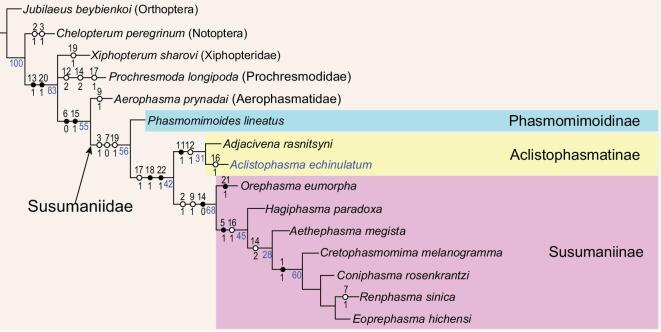
Phylogenetic analysis of Susumaniidae based on wing venation, the strict consensus tree, tree length = 41 steps, consistency index (CI) = 0.60, retention index (RI) = 0.79. The numbers under the branch nodes are bootstrap support values (data in blue). (•) Unambiguous unique characteristics; (°) Homoplasious characteristics.

Hitherto, the Middle Jurassic stick insect, *Adjacivena rasnitsyni* from Inner Mongolia, China, represented the earliest fossil occurrence of Susumanioidea [24]. The new species, *Aclistophasma echinulatum*, from the same locality, adds an additional occurrence of the superfamily and, along with *A. rasnitsyni*, constitutes a new subfamilial clade: Aclistophasmatinae, within Susumaniidae (Fig. 3). Previously, Susumaniidae were divided into two subfamilies—Susumaniinae and Phasmomimoidinae [25]. Our phylogenetic analysis based on characteristics of the wing venation recovered three clades of Susumaniidae (Fig. 3), i.e. Phasmomimoidinae, Susumaniinae and the new subfamilial clade described herein. Furthermore, the phylogenetic analysis indicates that Phasmomimoidinae are the earliest diverging group of Susumaniidae, mainly owing to the possession of a large number of plesiomorphies in the hindwings of these genera. The hindwing of Phasmomimoidinae has three RP branches, while Susumaniinae and Aclistophasmatinae have a single branch of RP that fuses with MA. Susumaniinae and Aclistophasmatinae also both have CuA+CuP fused apically in the hindwings, which differs from the unfused CuA and CuP in Phasmomimoidinae. Aclistophasmatinae were recovered as a sister to Susumaniinae. Aclistophasmatinae can easily be distinguished from Susumaniinae by the forking of M proximal to the origin of RP (*vs.* M forking apical to the origin of RP in Susumaniinae).

**Figure 4.**
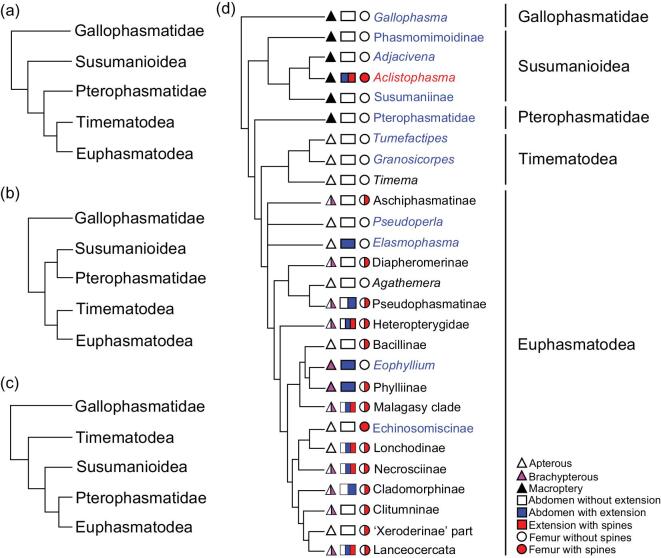
Phylogeny of Phasmatodea, with characteristics of the wings, abdominal extensions and femoral spines mapped. The phylogenies (a), (b) and (c) are the supposed evolutionary patterns of Phasmatodea. The phylogeny (d) same as (a) is modified from the analyses of Yang *et al.* (2019) and Simon *et al.* (2019). Fossil taxa (blue and red fonts) and modern groups (black fonts) are also shown.

The taxonomic placement of some Mesozoic winged Phasmatodea remains controversial based on wing venation alone. Five extinct families (Prochresmodidae, Xiphopteridae, Aeroplanidae, Cretophasmatidae and Aerophasmatidae) are considered to be closely related to Phasmatodea rather than Orthoptera [26–28]. A phylogenetic analysis including these taxa (Supplementary Fig. 1a) suggests that these families have a close relationship with other winged stick insects, and they share the characters such as: MP of forewing and hindwing simple and Cu of hindwing with two branches. These characteristics are probably synapomorphies of winged stick insects. In this analysis (Supplementary Fig. 1a), Susumanioidea were recovered as a sister to crown-group stick insects, although other fossil groups that could have affinities even closer to Euphasmatodea, such as Pterophasmatidae, were not included in this secondary analysis [29]. Nonetheless, from our exploratory analysis, synapomorphies of Susumanioidea and extant stick insects included the lack of a forewing precostal area, a simple RP in the hindwing (except Phasmomimoidinae), 2–7A of the hindwing with a common origin at the wing base, presence of abdominal tergal extensions, pentamerous tarsi, presence of a vomer on male abdominal segment X and unsegmented cerci. From this body of evidence, it is apparent that Susumanioidea are closely related to crown-group Phasmatodea and are a derived part of the grade leading to the Timematodea + Euphasmatodea clade. It is supposed that there are three possible evolutionary patterns of Phasmatodea, as shown in Fig. 4a–c. However, according to the undisputed synapomorphy of metasternum distinctly separated from abdominal sternum I in Susumanioidea (*vs*. metasternum fused with abdominal sternum I in Pterophasmatidae, Timematodea and Euphasmatodea), the hypothesis of Fig. 4a and d is most likely. *Gallophasma* from the Eocene of France with elongate maxillary palpi, expanded pronotal lateral sides, well-developed ovipositor and tetramerous cerci is controversially assigned to Phasmatodea. Nonetheless, its forewing venation is similar to that of Susumanioidea: the metatergum is fused with abdominal tergum I—a feature also present in Euphasmatodea (*vs*. metatergum distinctly separated from abdominal tergum I in Susumanioidea and Timematodea, unclear in Pterophasmatidae) [30,31]. Therefore, it is possible that *Gallophasma* could have a closer relationship with Phasmatodea rather than within the Susumanioidea + Pterophasmatidae + Timematodea + Euphasmatodea clade.

### The evolution of abdominal extensions and femoral spines in Phasmatodea

Modern stick and leaf insects have a unique abdominal structure, sometimes with extensions that may aid their crypsis. Among stick insects with abdominal extensions, most have only the terga modified in this way, while, in the leaf insects (Phylliinae), both the terga and sterna are extended [32]. Not surprisingly, the Eocene leaf insect *Eophyllium messelensis* had both the terga and sterna extended as in their modern counterparts [21]. Among Mesozoic taxa, the mid-Cretaceous *Elasmophasma stictum* exhibited traces of multiple tergal extensions, analogous to many modern stick insects with similar modifications [20]. Interestingly, *Aclistophasma echinulatum* from the Middle Jurassic has these same abdominal modifications and therefore represents the earliest occurrence of such abdominal extensions. The abdominal terga of *A. echinulatum* were laterally extended and each segment was distinct from those adjacent (Fig. 2a and f), but the sterna and pleura were not modified (Fig. 2b, g and h). The extensions of each abdominal tergum in *A. echinulatum* were convex and often apicolaterally projected with acutely rounded apices and bore numerous minute spines or spicules along the margins (Fig. 2b and d). Among extant Phasmatodea, the extensions are often produced on the thorax, abdomen and legs. For example, the leaf insect *Phyllium fallorum* has lateral extensions on both sides of the femora and tibiae, pleurae of the thorax, as well as the abdominal terga and sterna [33]. However, in *A. echinulatum*, extensions are only present on the abdominal terga. This is perhaps not surprising given that pleural and sternal modifications are only found in more derived clades (e.g. Phylliinae) and among taxa that are significantly younger in age. Tergal modifications clearly predate subsequent additional sclerotic extensions.

Femoral spines are usually used as defensive structures among Phasmatodea and are most greatly developed in males, and less so or even absent in females. The legs are flexed at the femoro-tibial joint and can force the femoral spines into an aggressor when the stick insect is restrained, ideally causing a potential predator to release its intended victim [1]. Similar spines were also present along the margins of the legs of *A. echinulatum* (Fig. 1g and h). Additionally, there is a spiny ridge medially along the upper surface of the femora. These spines are structurally similar to those of extant stick insects and assuredly functioned in an identical fashion. While some modern Phasmatodea may have spines distributed widely over the femora, tibiae, thorax and even the abdomen, the spines of *A. echinulatum* were restricted to the femora. Interestingly, when exploring the phylogenetic distribution of abdominal extensions and femoral spines among Phasmatodea, it is clear that these have evolved multiple times within the clade (Fig. 4d)—a pattern in agreement with results from analyses of extant taxa based on morphological and molecular data [29,34–39].

### Early mimetic and defensive strategies for Jurassic stick insects

**Figure 5.**
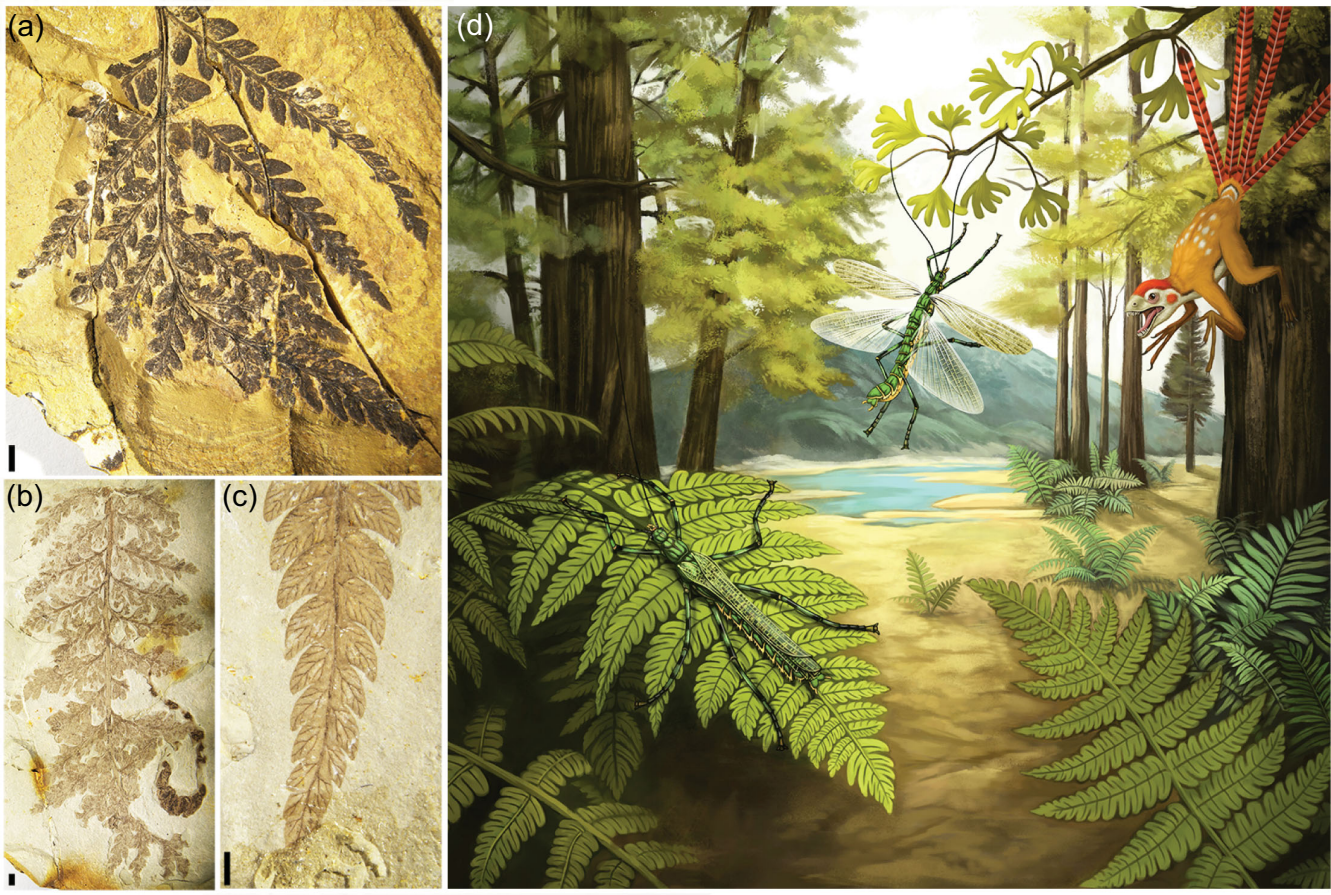
A reconstruction of *Aclistophasma echinulatum* in its presumed contemporaneous surroundings and potential plants. (a) Frond of *Cladophlebis* sp. (Osmundales: Osmundaceae). (b) Frond of *Coniopteris* sp. (Cyatheales: Dicksoniaceae). (c) Leaf of *Cladophlebis* sp. (d) Three-dimensional ecological reconstruction of *Aclistophasma echinulatum* gen. et sp. nov. Scale bars: (a)–(c), 2 mm.

*Aclistophasma echinulatum* possesses a combination of characteristics associated with mimicry and active defense, including a large body size, large wings, extended abdominal terga with marginal spicules and distinct femoral spination. It is clear that, by the Middle Jurassic, at least some stick insects had evolved passive and active antipredator defenses. The form of the tergal extensions was similar to the overall form of various leaves from ferns that coexisted in the same deposits and that of a comparable size and shape (Fig. 5a–c). Such a close approximation likely provided the insect with improved mimicry if motionless among such ferns, permitting it to avoid detection by predators. Although the large body size and covering of the wings might have been negative factors against concealment, they would have been beneficial as part of a secondary defensive suite. The large body size, which would give the appearance of increasing significantly if the large wings were spread, would potentially permit the insect to evade or dissuade smaller predators. More importantly, the wings would also have allowed *A. echinulatum* to simply take flight in order to avoid a potential predator after detection, particularly any that were not flight-capable themselves. The femoral spines, functioning like those of modern stick insects (Fig. 2c), would have presumably also allowed *A. echinulatum* to defend itself to a limited degree when seized [1]. The femoral spines of *A. echinulatum* were shorter and less numerous than those of modern species such as *Oncotophasma martini*, suggesting a limited ability to counterattack on the part of the Jurassic species. However, the combination of the femoral spines curved, spinulose margins to the tergal extensions would have made the overall insect rather prickly when grasped. Among living stick insects, curved tips and spines or spicules are commonly found on the abdominal extensions (e.g. *Heteropteryx dilatata* (Fig. 2c, e and h) and *Extatosoma tiaratum*) and these enhance mimicry with leaves, mosses or lichens, as well as providing some degree of defense [40,41]. The same was likely true for *A. echinulatum*, with the extensions serving both as defensive armature as well as supplementing the overall crypsis.

Most extant stick insects spend their lives sitting in trees and bushes, where they feed on foliage, often resting motionless to avoid detection by predators [3]. Although, today, most Phasmatodea feed on angiosperms, there are taxa who live and feed upon gymnosperms, such as conifers and firs [42]. A small number of more specialized feeders, such as the popular Peruvian fern stick insect, feed on brackens and a range of wild and cultivated ferns [43]. The Early Cretaceous stick insect *Cretophasmomima melanogramma* was an apparent mimic of ginkgoes and likely fed upon these same plants [44]. Ferns and gymnosperms predominated in the Jurassic Yanliao Biota [7,45] and *A. echinulatum* lived and fed within this flora, apparently mimicking at least some ferns and perhaps gymnosperms, such as *Coniopteris* (Fig. 5b), *Cladophlebis* (Fig. 5a and c) and early ginkgoes in this deposit [45]. Potential predators were abundant and diverse within the same fauna, including diverse insectivorous vertebrates and spiders [46–48] (Fig. 5d). With such a rich array of predators, analogous to those today preying upon Phasmatodea, early stick insects would have had much the same pressures for survival as their modern relatives. Although the mimicry observed in *A. echinulatum* was quite different and evolved for a different flora, the morphological and perhaps behavioral tools used to build up their defense, albeit to a lesser development, were analogous to those that persist today.

## MATERIALS AND METHODS

### Specimen imaging and terminology

The material described here is housed in the Key Lab of Insect Evolution and Environmental Changes, College of Life Sciences, Capital Normal University, Beijing, China (CNUB; Dong Ren, Curator). The specimen CNU-PHA-NN2019006 was examined under a Leica M205C dissecting microscope. All photographs were taken with a Nikon SMZ 25 microscope with an attached Nikon DS-Ri2 digital camera system and a Nikon ECLIPSE Ni microscope with an attached Nikon DS-Ri2 digital camera system. Line drawings were prepared using Adobe Illustrator CC and Adobe Photoshop CC graphics software.

The wing-venation nomenclature follows Wang *et al.* (2014) [44]. The following abbreviations have been used throughout: 1A, the first anal vein; Cu, cubitus; CuA, cubital anterior; CuP, cubital posterior; CuPa, anterior branch of CuP; CuPaα, anterior branch of CuPa; CuPaβ, posterior branch of CuPa; CuPb, posterior branch of CuP; M, media; MA, medial anterior; MP, medial posterior; R, radius; RA, radial anterior; RP, radial posterior; ScA, subcostal anterior; ScP, subcostal posterior.

### Phylogenetic analysis

We carried out a phylogenetic analysis by using wing-venation characteristics to confirm the taxonomic position of the new taxon, because most of the extinct winged Phasmatodea lack preserved body features. For the phylogenetic analysis of Susumanioidea (Fig. 3), we chose 10 species of Susumanioidea (*Hagiphasma paradoxa, Orephasma eumorpha, Aethephasma megista, Adjacivena rasnitsyni, Cretophasmomima melanogramma, Renphasma sinica, Phasmomimoides lineatus, Coniphasma rosenkrantzi, Eoprephasma hichensi* and *Aclistophasma echinulatum*) as the in-group and *Jubilaeus beybienkoi* (Orthoptera), *Chelopterum peregrinum* (Notoptera), *Xiphopterum sharovi* (Xiphopteridae), *Prochresmoda longipoda* (Prochresmodidae) and *Aerophasma prynadai* (Aerophasmatidae) as the out-groups. A total of 23 wing-venation characteristics are listed in Supplementary Table 1 and the characteristic–state matrix consisting of 15 taxa and 23 characteristics is provided in Supplementary Table 2.

We also carried out a phylogenetic analysis using wing-venation characteristics to confirm the taxonomic position of Susumanioidea and the relationship among Susumanioidea, Prochresmodidae, Xiphopteridae, Aeroplanidae, Cretophasmatidae, Aerophasmatidae and extant stick insects. For the phylogenetic analysis (Supplementary Fig. 1a), we chose nine genera of Mesozoic extinct groups (*Xiphopterum, Prochresmoda, Palaeochresmoda, Paraplana, Aerophasma, Jurophasma, Cretophasma, Orephasma* and *Aclistophasma*) and two species of extant stick insects (*Heteropteryx dilatata, Paracyphocrania major*) as in-groups, with *Jubilaeus* (Orthoptera) and *Chelopterum* (Notoptera) as out-groups. A total of 32 wing-venation characteristics are listed in Supplementary Table 3 and the characteristic–state matrix consisting of 13 taxa and 32 characteristics is provided in Supplementary Table 4.

Parsimony analyses were performed using WinClada (Version 1.00.08) [49] and NONA (Version 2.0) [50]. Tree search implemented a heuristic search method and the options were set to hold 10 000 trees, 1000 replications, 100 starting tree replications and a multiple TBR + TBR search strategy. All characteristics were considered unordered and weighted equally. Bootstrap supporting values were determined by using NONA with 1000 replications and are represented as numbers under the branch nodes.

Nomenclatural acts established herein are registered in ZooBank (www.zoobank.org) following the requirements of the International Code of Zoological Nomenclature and listed under LSID: urn: lsid: zoobank.org: pub: D982793C-CD0A-4121-B1C7–1F55C8A15DF3 (for publication); LSID: urn: lsid: zoobank.org: act: 377263D2-AD21–4D36-BB04–307ACDE61231 (for Aclistophasmatinae subfam. nov.); LSID: urn: lsid: zoobank.org: act: D76D4CDA-B924–438B-9648–78B20A12BD78 (for *Aclistophasma* gen. nov.); LSID: urn: lsid: zoobank.org: act: 6EC04B10-BFE0–441D-B562–420CAB8D83DB (for *Aclistophasma echinulatum* sp. nov.).

## Supplementary Material

nwaa056_Supplymentary_Files
