# Supplementary material for: Early specializations for mimicry and defense in a Jurassic stick insect
Source: Natl Sci Rev. 2020 Apr 2;8(1):nwaa056. doi: 10.1093/nsr/nwaa056 (PMC8288419; doi:10.1093/nsr/nwaa056)
Supplement: nwaa056_Supplymentary_Files [file nwaa056_supplymentary_files.zip › Stick_Insect_SI_03292020.docx]

**Supplementary Online Content**

**Early specializations for mimicry and defense in a Jurassic stick insect**

**Hongru Yang, Chaofan Shi,** **Michael S. Engel, Zhipeng Zhao, Dong Ren*, Taiping Gao***

**Supplementary Text and References.**

**Supplementary Figure S1.** Phylogenetic analysis of winged stick insects based on wing venation.

**Supplementary Table S1.** Definition of characters and character states of figure 3.

**Supplementary Table S2.** Character state matrix of 23 characters for the 16 taxa included in the phylogenetic study of figure 3.

**Supplementary Table S3.** Definition of characters and character states of figure S1a.

**Supplementary Table S4.** Character state matrix of 32 characters for the 13 taxa included in the phylogenetic study of figure S1a.

**Supplementary Text**

**Subfamily Aclistophasmatinae Yang, Engel & Gao subfam. nov.**

**Remarks.**

Susumanioidea include following genera: *Palaeopteron* Rice, 1969 (Upper Cretaceous, Labrador, Canada), *Coniphasma* Birket-Smith, 1981 (Upper Cretaceous, Greenland), *Promastacoides* Kevan & Wighton, 1981 (Paleocene, south-central Alberta, Canada), *Phasmomimella* Kevan & Wighton, 1981 (Paleocene, south-central Alberta, Canada), *Cretophasmomima* Kuzmina, 1985 (Lower Cretaceous, Baissa, Siberia), *Paraphasmomimella* Kuzmina, 1985 (Lower Cretaceous, Baissa, Siberia), *Eosusumania* Gorochov, 1988 (Lower Cretaceous, Siberia), *Prosusumania* Gorochov, 1988 (Lower Cretaceous, Siberia), *Cretophasmomimoides* Gorochov, 1988 (Lower Cretaceous, Siberia), *Susumania* Gorochov, 1988 (Upper Cretaceous, Siberia), *Kolymoptera* Gorochov, 1988 (Upper Cretaceous, Siberia), *Hagiphasma* Ren, 1997 (Lower Cretaceous, Liaoning Province, China), *Aethephasma* Ren, 1997 (Lower Cretaceous, Hebei Province, China), *Orephasma* Ren, 1997 (Lower Cretaceous, Hebei Province, China), *Phasmomimula* Kevan & Wighton, 1981 (Paleocene, south-central Alberta, Canada), *Phasmomimoides* Sharov, 1968 (Upper Jurassic, Karatau, Kazakhstan) [1,2], *Renphasma* Nel & Delfosse, 2011 (Lower Cretaceous, Liaoning Province, China) [3], *Adjacivena* Shang, Béthoux & Ren, 2011 (Middle Jurassic, Inner Mongolia, China) [4] and *Eoprephasma* Archibald & Bradler, 2015 (Early Eocene, British Columbia, Canada) [5].

**References**

1. Ren D. First record of fossil stick-insects from China with analyses of some paleobiological features (Phasmatodea: Hagiphasmatidae fam. nov.). *Acta Zootaxon. Sin*. 1997; **22**: 268–282 (in Chinese).
2. Gorochov AV. Phasmomimidae: are they Orthoptera or Phasmatoptera? *Paleontol. J*. 2000; **34**: 295–300.
3. Nel A, Delfosse E. A new Chinese Mesozoic stick insect. *Acta Palaeontol. Pol*. 2011; **56**: 429–432.
4. Shang LJ, Béthoux O, Ren D. New stem-Phasmatodea from the Middle Jurassic of China. *Eur. J. of Entomol*. 2011; **108**: 677–685.
5. Archibald SB, Bradler S. Stem-group stick insects (Phasmatodea) in the early Eocene at McAbee, British Columbia, Canada, and Republic, Washington, United States of America. *Can. Entomol.* 2015; **147**: 744–753.

**Supplementary Figure S1.** Phylogenetic analysis of winged stick insects based on wing venation. (a) The strict consensus tree, tree length = 62 steps, consistency index (CI) = 0.61, retention index (RI) = 0.71. (b) Wing venation of *Prochresmoda longipoda* Sharov, 1968. (c) Wing venation of *Xiphopterum sharovi* Gorochov, 1994. (d) *Paraplana affinis* Sharov, 1968. (e) *Cretophasma raggei* Sharov, 1968. (f) *Aerophasma prynadai* Martynov, 1928. (g) *Aclistophasma echinulatum* gen. et sp. nov. (h) Male of *Heteropteryx dilatata* Parkinson, 1798. (i) *Paracyphocrania major* Hennemann, Conle, & Suzuki, 2015. Green rectangle highlights extant winged stick insects.


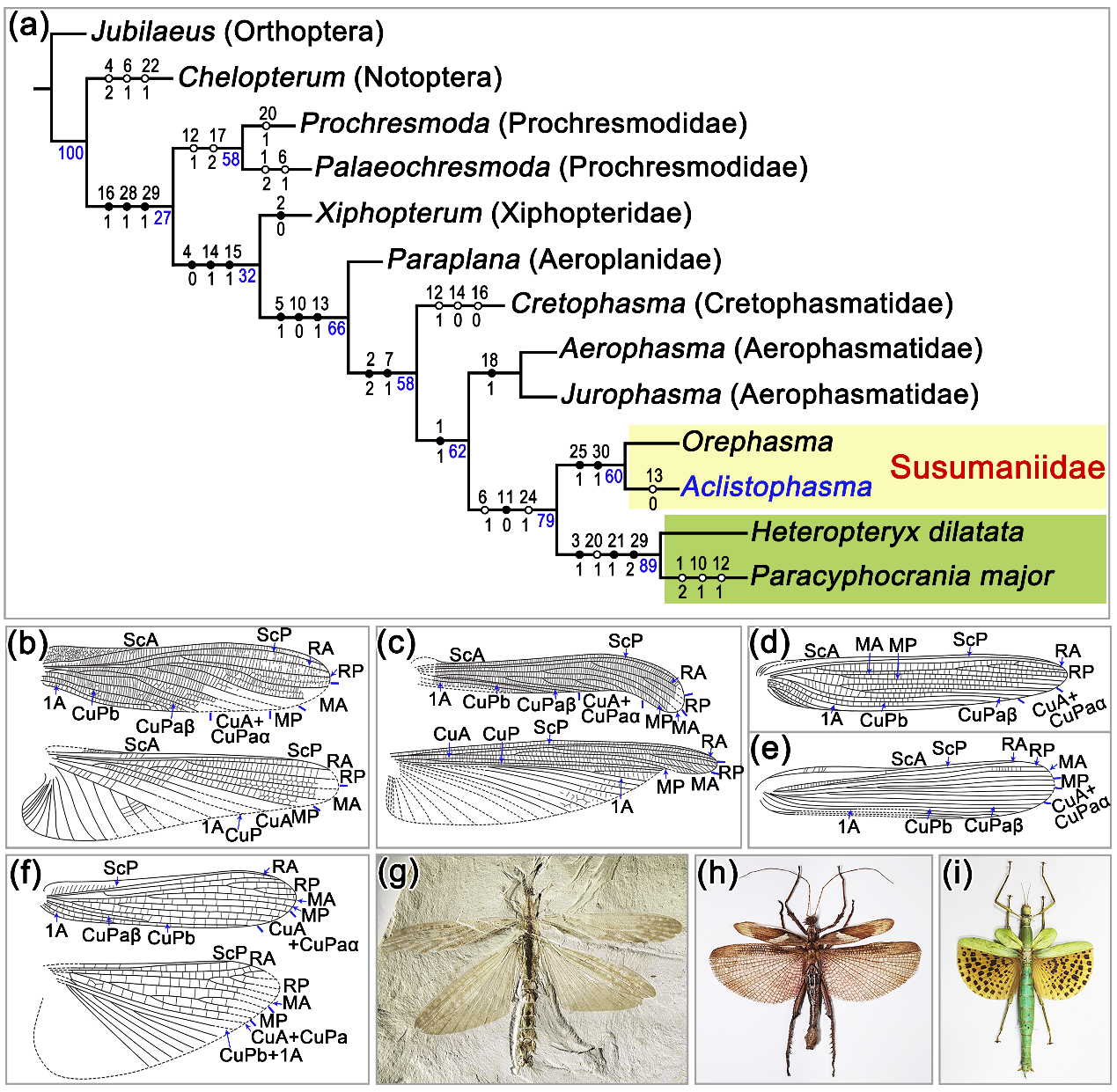


**Supplementary Table S1.** Definition of characters and character states of figure 3.

No. Characters and character states

1. Crossveins of forewing and hind wing: 0 - dense and close to each other; 1 - few and far away from each other.
2. Aspect ratio of forewing: 0 - ≥4; 1 - <4.
3. Precostal area of forewing: 0 - present; 1 - absent.
4. Precostal area of forewing: 0 - wider than area between ScA and ScP; 1- narrower than area between ScA and ScP.
5. End of ScP: 0 - apical on forewing; 1 - medial or proximal on forewing.
6. Branch location of R: 0 - proximal part of forewing; 1 - medial or posterior part of forewing.
7. First branch location of RP of forewing: 0 - proximal of RP origin; 1 - apical of RP origin.
8. Branches of RP of forewing: 0 - ≥3; 1 - <3.
9. Branch location of M: 0 - in front of RP origin; 1 - behind of RP origin.
10. Branches of M of forewing: 0 - >2; 1 - =2.
11. MP of forewing: 0 - not approaching CuA; 1 - approaching CuA.
12. Branches of CuA+CuPa of forewing: 0 - >3; 1 - =3; 2 - <3.
13. Anal veins of forewing: 0 - >2; 1 - ≤2.
14. Anal area in proximal part: 0 - wide, bulgy; 1 - narrow, not bulgy; 2 - sunken.
15. Anal fold at edge of hind wing: 0 - sunken; 1 - smooth.
16. Terminus of ScP: 0 - apical on hind wing; 1 - medial or proximal on hind wing.
17. RP of hind wing: 0 - branched; 1 - simple.
18. RP+MA in hind wing: 0 - not fused; 1- fused.
19. Branches of M of hind wing: 0 - >2; 1 - =2.
20. Branches of Cu of hind wing: 0 - >2; 1 - =2.
21. MP terminating into CuP: 0 - absent; 1 - present.
22. CuA+CuP fused apically on hind wing: 0 - absent; 1 - present.
23. 2–7A of hind wing: 0 - without a common origin at wing base; 1 - with a common origin at wing base.

**Supplementary Table S2.** Character state matrix of 23 characters for the 16 taxa included in the phylogenetic study of figure 3.

| Taxa/ character | 1 | 2 | 3 | 4 | 5 | 6 | 7 | 8 | 9 | 10 | 11 | 12 | 13 | 14 | 15 | 16 | 17 | 18 | 19 | 20 | 21 | 22 | 23 |
| --- | --- | --- | --- | --- | --- | --- | --- | --- | --- | --- | --- | --- | --- | --- | --- | --- | --- | --- | --- | --- | --- | --- | --- |
| *Jubilaeus beybienkoi* (Orthoptera) | 0 | 0 | 0 | 0 | 0 | 1 | 1 | 0 | 0 | 0 | 0 | 0 | 0 | 1 | 0 | 0 | 0 | 0 | 0 | 0 | 0 | 0 | 0 |
| *Chelopterum peregrinum* (Notoptera) | 0 | 1 | 1 | – | 0 | 1 | 1 | 0+1 | 0 | 0 | 0 | 0 | 0 | 1 | 0 | 0 | 0 | 0 | 0 | 0 | 0 | 0 | 0 |
| *Xiphopterum sharovi* (Xiphopteridae) | 0 | 0 | 0 | 0 | 0 | 1 | 1 | 1 | 0 | 1 | 0 | 0 | 1 | 1 | 0 | 0 | 0 | 0 | 1 | 1 | 0 | 0 | ? |
| *Prochresmoda longipoda* (Prochresmodidae) | 0 | 0 | 0 | 0 | 0 | 1 | – | 1 | 0 | 0 | 0 | 2 | 1 | 2 | 0 | 0 | 1 | 0 | 0 | 1 | 0 | 0 | ? |
| *Aerophasma prynadai* (Aerophasmatidae) | 0 | 0 | 0 | 1 | 0 | 0 | 1 | 0 | 1 | 1 | 0 | 0 | 1 | 1 | 1 | 0 | 0 | 0 | 0 | 1 | 0 | 0 | ? |
| *Hagiphasma paradoxa* | 0 | 1 | 1 | – | 1 | 0 | 0 | 1 | 1 | 1 | 0 | 0 | 1 | 0 | 1 | 1 | 1 | 1 | 1 | 1 | 0 | 1 | 1 |
| *Orephasma eumorpha* | 0 | 1 | 1 | – | 0 | 0 | 0 | 1 | 1 | 1 | 0 | 0 | 1 | 0 | 1 | 0 | 1 | 1 | 1 | 1 | 1 | 1 | 1 |
| *Aethephasma megista* | 0 | 1 | 1 | – | 1 | 0 | 0 | 1 | 1 | 1 | 0 | 0 | 1 | 2 | 1 | 1 | 1 | 1 | 1 | 1 | 0 | 1 | 1 |
| *Adjacivena rasnitsyni* | 0 | 0 | 1 | – | 0 | 0 | 0 | 0 | 0 | 1 | 1 | 1 | 1 | 1 | 1 | 0 | 1 | 1 | 1 | 1 | ？ | ？ | ？ |
| *Cretophasmomima melanogramma* | 1 | 1 | 1 | – | 1 | 0 | 0 | 1 | 1 | 1 | 0 | 2 | 1 | 2 | 1 | 1 | 1 | 1 | 1 | ？ | ？ | ？ | ？ |
| *Aclistophasma echinulatum* | 0 | 0 | 1 | – | 0 | 0 | 0 | 1 | 0 | 1 | 1 | 1 | 1 | 1 | 1 | 1 | 0+1 | 1 | 1 | 1 | 0 | 1 | 1 |
| *Renphasma sinica* | 1 | 1 | 1 | – | 1 | 0 | 1 | 1 | 1 | 1 | 0 | 1 | 1 | 2 | ？ | ？ | ? | ? | ? | ? | ? | ? | ? |
| *Phasmomimoides lineatus* | 0 | 0 | 1 | – | 0 | 0 | 0 | 0 | 0 | 1 | 0 | 0+1 | 1 | 1 | 1 | 0 | 0 | 0 | 1 | ? | 0 | 0 | ? |
| *Coniphasma rosenkrantzi* | 1 | 1 | 1 | – | 1 | 0 | 0 | 1 | 1 | 1 | 0 | 1 | 1 | 2 | ？ | ？ | ? | ? | ? | ? | ? | ? | ? |
| *Eoprephasma hichensi* | 1 | 1 | 1 | – | 1 | 0 | 0 | 1 | 1 | 1 | 0 | 1 | 1 | 2 | ？ | ？ | ? | ? | ? | ? | ? | ? | ? |

**Supplementary Table S3.** Definition of characters and character states of figure S1a.

No. Characters and character states

1. Crossveins of forewing and hind wing: 0 - dense and close to each other; 1 - dense but distant from each other; 2 - few and far away from each other.
2. Forewing and hind wing: 0 - apex recurved; 1 - apex not curved, narrower than medial part; 2 - apex not curved, wider than or equal to medial part.
3. Forewing shorted: 0 - absent; 1 - present.
4. Aspect ratio of forewing: 0 - >5; 1 - ≤5 and ≥4; 2 - <4.
5. Sunken at terminus of ScA of forewing: 0 - present; 1 - absent.
6. Precostal area of forewing: 0 - present; 1 - absent.
7. Terminus of ScA: 0 - proximal on forewing; 1 - medial on forewing.
8. Precostal area of forewing: 0 - wider than area between ScA and ScP; 1 - equal to area between ScA and ScP; 2 - narrower than area between ScA and ScP.
9. Area between ScA and ScP in proximal part: 0 - narrower than or equal to posterior part; 1 - wider than posterior part.
10. Branch location of R: 0 - in proximal part of forewing; 1 - in medial or posterior part of forewing.
11. First branch location of RP of forewing: 0 - proximal of RP origin; 1 - apical of RP origin.
12. Branches of RP of forewing: 0 - ≥2; 1 - <2.
13. Branch location of M: 0 - in front of RP origin; 1 - behind RP origin.
14. Branches of M of forewing: 0 - >2; 1 - =2.
15. MA of forewing: 0 - branched; 1 - simple.
16. MP of forewing: 0 - branched; 1 - simple.
17. Branches of CuA+CuPa of forewing: 0 - >3; 1 - =3; 2 - <3.
18. CuPb ending on 1A: 0 - absent; 1 - present.
19. Anal veins of forewing: 0 - ≥3; 1 - <3.
20. Anal area in proximal part: 0 - wide; 1 - narrow, sunken.
21. Remigium of hind wing: 0 - wide; 1 - narrow.
22. Apical angle of hind wing: 0 - sharp; 1 - rounded.
23. Anal fold at edge of hind wing: 0 - sunken; 1 - smooth.
24. Branches of RP of hind wing: 0 - ≥2; 1 - <2.
25. RP+MA in hind wing: 0 - not fused; 1- fused.
26. Branches of M of hind wing: 0 - >2; 1 - =2.
27. MA of hind wing: 0 - branched; 1 - simple.
28. MP of hind wing: 0 - branched; 1 - simple.
29. Branches of Cu of hind wing: 0 - >2; 1 - =2; 2 - <2.
30. CuA+CuP apically on hind wing: 0 – not fused; 1 - fused.
31. Cu/CuP+1A of hind wing: 0 - not fused; 1 - adjoining or fused.
32. 2–7A of hind wing: 0 - without a common origin at wing base; 1 - with a common origin at wing base.

**Supplementary Table S4.** Character state matrix of 32 characters for the 13 taxa included in the phylogenetic study of figure S1a.

| Taxa/ character | 1 | 2 | 3 | 4 | 5 | 6 | 7 | 8 | 9 | 10 | 11 | 12 | 13 | 14 | 15 | 16 | 17 | 18 | 19 | 20 | 21 | 22 | 23 |
| --- | --- | --- | --- | --- | --- | --- | --- | --- | --- | --- | --- | --- | --- | --- | --- | --- | --- | --- | --- | --- | --- | --- | --- |
| *Jubilaeus* (Orthoptera) | 0 | 1 | 0 | 1 | 0 | 0 | 0 | 0 | 0 | 1 | 1 | 0 | 0 | 0 | 0 | 0 | 0 | 0 | 0 | 0 | 0 | 0 | 0 |
| *Chelopterum* (Notoptera) | 0 | 1 | 0 | 2 | – | 1 | – | – | 1 | 1 | 1 | 0 | 0 | 0 | 0+1 | 0 | 0 | 0 | 0 | 0 | 0 | 1 | 0 |
| *Xiphopterum* (Xiphopteridae) | 0 | 0 | 0 | 0 | 0 | 0 | 0 | 0 | 0 | 1 | 1 | 0 | 0 | 1 | 1 | 1 | 0 | 0 | 1 | 0 | 0 | 0 | 0 |
| *Prochresmoda* (Prochresmodidae) | 0 | 1 | 0 | 1 | 0 | 0 | 0 | 0 | 0 | 1 | – | 1 | 0 | 0 | 0 | 1 | 2 | 0 | 1 | 1 | 0 | 0 | 0 |
| *Palaeochresmoda* (Prochresmodidae) | 2 | 1 | 0 | 1 | – | 1 | – | – | 1 | 1 | – | 1 | 0 | 0 | 0 | 1 | 2 | 0 | 1 | 0 | ? | ? | ? |
| *Paraplana* (Aeroplanidae) | 0 | 1 | 0 | 0 | 1 | 0 | 0 | 1 | 1 | 0 | 1 | 0 | 1 | 1 | 1 | 1 | 0 | 0 | 0 | 0 | ? | ? | ? |
| *Aerophasma* (Aerophasmatidae) | 1 | 2 | 0 | 1 | 1 | 0 | 1 | 2 | 1 | 0 | 1 | 0 | 1 | 1 | 1 | 1 | 0 | 1 | 1 | 0 | 0 | 1 | 1 |
| *Jurophasma* (Aerophasmatidae) | 1 | 2 | 0 | 1 | 1 | 0 | 1 | 2 | 1 | 0 | 1 | 0 | 1 | 1 | 1 | 1 | 0 | 1 | 1 | 0 | ? | ? | ? |
| *Cretophasma* (Cretophasmatidae) | 0 | 2 | 0 | 0 | 1 | 0 | 1 | 2 | 1 | 0 | – | 1 | 1 | 0 | 1 | 0 | 0 | 0 | 0 | 0 | ? | ? | ? |
| *Orephasma* (Susumanioidea) | 1 | 2 | 0 | 2 | – | 1 | – | – | 1 | 0 | 0 | 0 | 1 | 1 | 1 | 1 | 1 | 0 | 1 | 0 | 0 | 1 | 1 |
| *Aclistophasma* (Susumanioidea) | 1 | 2 | 0 | 1 | – | 1 | – | – | 1 | 0 | 0 | 0 | 0 | 1 | 1 | 1 | 1 | 0 | 1 | 0 | 0 | 1 | 1 |
| *Heteropteryx dilatata* | 1 | 2 | 1 | 2 | – | 1 | – | – | 1 | 0 | 0 | 0 | 1 | 1 | 1 | 1 | 2 | 0 | 1 | 1 | 1 | 1 | 1 |
| *Paracyphocrania major* | 2 | 2 | 1 | 2 | – | 1 | – | – | 1 | 1 | – | 1 | 1 | 1 | 1 | 1 | 2 | 0 | 1 | 1 | 1 | 1 | 1 |

| Taxa/ character | 24 | 25 | 26 | 27 | 28 | 29 | 30 | 31 | 32 |
| --- | --- | --- | --- | --- | --- | --- | --- | --- | --- |
| *Jubilaeus* (Orthoptera) | 0 | 0 | 0 | 0 | 0 | 0 | 0 | 0 | 0 |
| *Chelopterum* (Notoptera) | 0 | 0 | 0 | 1 | 0 | 0 | 0 | 0 | 0 |
| *Xiphopterum* (Xiphopteridae) | 0 | 0 | 1 | 1 | 1 | 1 | 0 | 0 | ? |
| *Prochresmoda* (Prochresmodidae) | 1 | 0 | 0 | 0 | 1 | 1 | 0 | 0 | ? |
| *Palaeochresmoda* (Prochresmodidae) | ? | ? | ? | ? | ? | ? | ? | ? | ? |
| *Paraplana* (Aeroplanidae) | ? | ? | ? | ? | ? | ? | ? | ? | ? |
| *Aerophasma* (Aerophasmatidae) | 0 | 0 | 0 | 1 | 0 | 0 | 0 | 1 | ? |
| *Jurophasma* (Aerophasmatidae) | ? | ? | ? | ? | ? | ? | ? | ? | ? |
| *Cretophasma* (Cretophasmatidae) | ? | ? | ? | ? | ? | ? | ? | ? | ? |
| *Orephasma* (Susumanioidea) | 1 | 1 | 1 | 1 | 1 | 1 | 1 | 1 | 1 |
| *Aclistophasma* (Susumanioidea) | 0+1 | 1 | 1 | 1 | 1 | 1 | 1 | 1 | 1 |
| *Heteropteryx dilatata* | 1 | 0 | 1 | 1 | 1 | 2 | 0 | 1 | 1 |
| *Paracyphocrania major* | 1 | 0 | 1 | 1 | 1 | 2 | 0 | 1 | 1 |
